# Role of metabolism in cancer cell radioresistance and radiosensitization methods

**DOI:** 10.1186/s13046-018-0758-7

**Published:** 2018-04-23

**Authors:** Le Tang, Fang Wei, Yingfen Wu, Yi He, Lei Shi, Fang Xiong, Zhaojian Gong, Can Guo, Xiayu Li, Hao Deng, Ke Cao, Ming Zhou, Bo Xiang, Xiaoling Li, Yong Li, Guiyuan Li, Wei Xiong, Zhaoyang Zeng

**Affiliations:** 1 The Key Laboratory of Carcinogenesis of the Chinese Ministry of Health, Xiangya Hospital, Central South University, Changsha, Hunan China; 2 The Key Laboratory of Carcinogenesis and Cancer Invasion of the Chinese Ministry of Education, Cancer Research Institute, Central South University, Changsha, Hunan China; 3 Hunan Key Laboratory of Nonresolving Inflammation and Cancer, Disease Genome Research Center, The Third Xiangya Hospital, Central South University, Changsha, Hunan China; 4 Hunan Key Laboratory of Translational Radiation Oncology, Hunan Cancer Hospital and the Affiliated Cancer Hospital of Xiangya School of Medicine, Central South University, Changsha, Hunan China; 5 Department of Cancer Biology, Lerner Research Institute, Cleveland Clinic, Cleveland, Ohio USA

**Keywords:** Radiotherapy, Radioresistance, Metabolic pathway, Sensitivity, Cancer

## Abstract

**Background:**

Radioresistance is a major factor leading to the failure of radiotherapy and poor prognosis in tumor patients. Following the application of radiotherapy, the activity of various metabolic pathways considerably changes, which may result in the development of resistance to radiation.

**Main body:**

Here, we discussed the relationships between radioresistance and mitochondrial and glucose metabolic pathways, aiming to elucidate the interplay between the tumor cell metabolism and radiotherapy resistance. In this review, we additionally summarized the potential therapeutic targets in the metabolic pathways.

**Short conclusion:**

The aim of this review was to provide a theoretical basis and relevant references, which may lead to the improvement of the sensitivity of radiotherapy and prolong the survival of cancer patients.

## Background

Cancer is a major health concern, and the conventional treatments for cancer include surgery, chemotherapy, targeted therapies, and immunotherapy. Since tumor cells show sensitivity to the ionizing radiation (IR), radiotherapy emerged as the main type of cancer treatment [1–3]. Radiotherapy directly induces DNA damage or indirectly induces the production of reactive oxygen species (ROS) in cancer cells. Additionally, radiotherapy combined with immunotherapy and chemotherapy may reverse tumor hypoxia by reducing tumor oxygen consumption,and alters tumor immune response, which may lead to considerable clinical improvements in many different types of tumor [4, 5]. Radiotherapy has the advantage of localized application, but the IR was shown to activate several epithelial-mesenchymal transition (EMT) transcription factors, including SNAI1, HIF1 (hypoxia inducible factor 1), ZEB1, and STAT3, promoting cancer cell metastasis [6]. Radioresistance leads to poor prognosis in cancer patients and it represents the main reason for radiotherapy failure, which can ultimately lead to tumor recurrence and metastases [7].

Cancer is closely associated with metabolic disorders [8–10]. Metabolic reprogramming, the alteration of the metabolic pathways in tumor cells during a response to hypoxia or malnutrition, is considered one of the hallmarks of cancer [11, 12]. Aberrant activation of oncogenes and tumor-related signaling pathways can induce the metabolic reprogramming of prostate or breast cancer cells, producing specific metabolic fingerprints [13, 14]. Furthermore, the inactivation of tumor suppressor genes is an important factor underlying tumor metabolic changes [15]. In contrast, metabolic changes can promote the development and progression of cancer as well [10]. Moreover, many studies confirmed that the metabolic syndrome, which includes obesity, cardiovascular diseases, and diabetes, has a profound impact on the occurrence and development of cancer [16–19]. As an important type 2 diabetes mellitus therapeutic, metformin showed efficacy against prostate cancer, breast cancer, and ovarian cancer [20–22], suggesting that the targeting of tumor metabolism may provide a new therapeutic strategy for cancer [23].

Metabolic changes can induce radioresistance as well [24], and the alterations in the glycolytic metabolism were shown to contribute to radioresistance development. Radiotherapy effects primarily depend on glucose metabolism [25, 26], while the mitochondrial metabolic alterations can be involved in this process as well. A comprehensive analysis of the metabolic pathways of cancer patients that underwent radiotherapy revealed an increased expression of genes that regulate mitochondrial functions, autophagy, and lysosomal degradation activities, as well as a strong reliance on mitochondrial respiration and diminished dependence on the Warburg effect [27]. Liu et al. [28] demonstrated that CDK1 mediates the activation of sirtuin 3 (SIRT3), regulates the mitochondrial protein deacetylation, thus promoting the metabolic balance, and enhances mitochondrial functions, inducing the anti-radiation effects in colon, glioma, and breast cancer cells.

Therefore, in this review, we discuss glucose and mitochondrial metabolisms as the main metabolic pathways involved in the radioresistance development. Additionally, we review several sensitizing agents targeting these pathways to enhance the radiosensitivity of cancer patients.

### Radioresistance of cancer cells

**Fig. 1:**
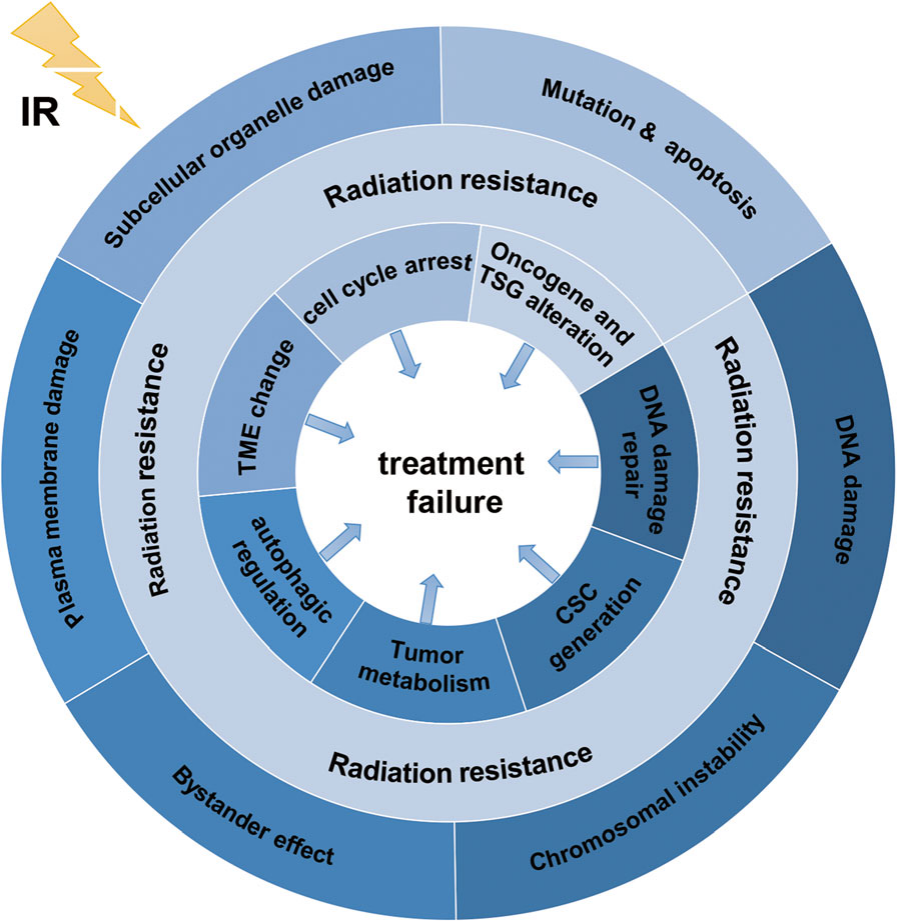
The biological effects of radiation and the mechanism of radiation resistance**.** The outer ring indicates the biological effects of IR under normal conditions. The abnormal alterations of these effects will further induce the occurrence of radiation resistance. The inner ring indicates the mechanism of radiation resistance and the biological changes in the occurrence of radioresistance. These abnormal changes are the important reasons for treatment failure of cancer patients

Since the discovery of the IR in 1895, radiotherapy emerged as the treatment-of-choice for many types of cancer, and has been applied as the first-line therapy for many human malignancies [29]. Tumor radiotherapy is a highly targeted and efficient method of destroying cancer cells that can lead to the curing of or palliation of many cancer patients after surgery. The IR induces oxidative stress in cancers cells [30], and free OH radicals are considered the IR-induced common mediators of DNA damage, including single-strand breaks (SSB) and double-strand breaks (DSB), which disturb the DNA structure, triggering cell death [31]. In addition to the DNA targeting, the IR can affect plasma membrane and subcellular organelles, and induce the activation of cell stress response-related genes and intracellular signaling pathways, triggering cell death [29]. Additionally, the irradiated cells may affect their non-irradiated neighbors through the bystander effect, or the transmission of the apoptotic signals to the surrounding unirradiated cells through a direct cellular contact or intercellular communication, which leads the unirradiated cells to exhibit similar biological effects to those of the irradiated cells [32]. Combined, these effects lead to the DNA damage, chromosomal instability, mutation and apoptosis in cancer cells, ultimately killing them (Fig. 1).

IR is the most effective therapeutic method for the treatment of many tumors; however, owing to radioresistance development, it remains only a conservative cancer treatment [33]. Radioresistance is a process in which the tumor cells or tissues adapt to the radiotherapy-induced changes and develop resistance to the IR. This is a complex process involving multiple genes, factors, and mechanisms [34, 35].

The mechanisms underlying the development of radioresistance have been the focus of many studies, and the main factors involved in this process were shown to be the following (Fig. 1):

1. DNA damage repair. Radiation can induce DNA damage response (DDR), which protects the cells against genomic instability, and the cells develop radioresistance by increasing the DDR rate. Following the detection of the IR-induced DNA damage in cancer cells, several main signaling pathways can rapidly respond and initiate DNA repair, including phosphatidylinositol 3-kinase (PI3K), mitogen-activated protein kinase (MAPK), and SIRT pathways [36, 37]. PI3K signaling pathway regulates the steady-state homologous recombination levels, promoting DNA damage repair, and the PI3K inhibitor PI-103 can significantly enhance radiation-induced death [38]. MAPK is the mediator of cellular stress responses, involved in the phosphorylation of XRCC1 and regulation of oxidative stress response, promoting the damage repair [39]. Furthermore, SIRT represents a class of histone deacetylases, and downregulation of SIRT1 promotes cell death by decreasing DNA repair enzyme levels, including MSH2, MSH6, and APEX1 [40].
2. Cell cycle arrest. Following the IR-induced DNA damage detection, molecules in the cell cycle checkpoints can regulate and arrest cell cycle progression, and 14–3-3σ, a member of 14–3-3 protein family, was shown to be closely associated with the radioresistance development by arresting cancer cells in the G2/M phase [41]. Moreover, tumor cells can utilize two distinct kinase signaling cascades for the DNA damage repair here, including ATM-Chk2 and ATR-Chk1 axes [42].
3. Oncogene and tumor suppressor alterations. For example, the cell adhesion molecule vitronectin (VTN) is an important oncogene, and the dysregulation of its expression promotes the migration and invasion of nasopharyngeal carcinoma (NPC) as well as resistance of the NPC cells to radiotherapy [43, 44]. Additionally, many miRNAs, e.g.*,* miR-29c and miR-22, have tumor-suppressor roles, and the alteration in their expression in lung and breast cancer cells represents an important cause of radioresistance [45, 46].
4. Changes in the tumor microenvironment (TME) may lead to the radioresistance development. Many immunosuppressive processes increase the risk of tumor recurrence and metastasis, and the immune evasion has emerged as a serious obstacle in cancer treatment [47]. Changes in the cytokine levels, EMT-related processes, and hypoxic conditions can promote radioresistance in tumor cells [48–51].
5. Autophagy. Autophagy is a metabolic-recycling pathway that involves a proteasome-independent degradation of cellular components [52]. Its dysfunctions may promote the development of systemic autoimmune diseases, such as lupus [53], while in cancer, it may promote or inhibit the survival and proliferation of cancer cells in the TME [54]. Temozolomide (TMZ) is an alkylating agent used to treat glioblastoma multiforme (GBM) and anaplastic astrocytomas, which induces autophagy and subsequent treatment resistance. When the transcription factor nuclear factor erythroid 2-related factor 2 (NRF2) inhibitor is used in combination with TMZ, a decrease in NRF2 expression increases TMZ-induced autophagy, attenuating cancer cell proliferation [55]. Chrysin, a NRF2 inhibitor, was shown to be able to overcome drug resistance by preventing the activation PI3K/AKT and ERK pathways [56]. P62 is a marker for degradation in autophagy, and its accumulation leads to the activation of NFΚB and stabilization of NRF2, which confers the resistance to hypoxic stress in tumor cells. Furthermore, autophagy preserves damaged organelles, including mitochondria [54]. In many cases, autophagy can reduce the rate of DNA damage-induced apoptosis, playing a protective role in tumor cells, which induces radioresistance in tumor cells [57, 58]. Targeting autophagy can be an effective way to improve the effects of radiotherapy [59].
6. The generation of cancer stem cells (CSCs) can represent a mechanism of resistance to radiotherapy. CSCs are undifferentiated cancer cells with high oncogenic activity, with the self-renewal ability and multi-directional differentiation potential [52]. CSCs tend to be responsible for the minimal residual disease (MRD), as they exhibit high metastatic potential after chemotherapy and radiation therapy. Furthermore, these cells are responsible for the development of tumor cell heterogeneity, which is a key factor in the resistance of anticancer therapy [52], and they are robust as well, including their cell cycle regulation, rapid response to DNA damage, detoxification or the mediation of cytotoxic agent efflux, anti-oxidative stress, ROS scavenging, and specific TME maintenance, which contribute to the development of radiation resistance [60–62]. Glioma stem cells are in contact with the endothelial cells in the perivascular niche, and display the hallmarks of radiation resistance [63]. The insulin-like growth factor (IGF) family was shown to be associated with the acquired or adaptive resistance of CSCs to the conventional anti-cancer therapies, including radiation therapy. Repeated irradiation induces the self-renewal potential of glioma stem cells by increasing IGF1 secretion and upregulating IGF type 1 receptor expression. Chronic receptor activation results in the inhibition of the PI3K-AKT signaling pathway, which in turn activates the transcription factor FOXO3A, leading to the cell cycle arrest. However, the acute irradiation of slow-circulating CSCs induces a rapid activation of IGF1-AKT signaling, which promotes radioprotection [64]. Chemotherapy was found to induce increased IGF2 expression, which paradoxically leads to the maintenance of dormant state in the osteosarcoma cells, promoting survival and conferring resistance to various treatments [65]. These results shown that the blocking of altered IGF signaling may represent a novel therapeutic approach to the selective treatment of glioma and osteosarcoma CSCs. The use of metformin, salinomycin, DECA-14, rapamycin, and other drugs may help prevent the development of radioresistant cells by inhibiting CSC self-renewal or redox capacity [52, 66].
7. Tumor metabolism. An increasing number of studies demonstrated that radioresistance is closely associated with the tumor metabolism alterations [24, 25]. Clinically, the main cause of radiotherapy failure is cellular radioresistance, conferred via glycolytic or mitochondrial metabolic changes [67]. Targeting cellular glucose or mitochondrial metabolism may improve the clinical response to cancer therapeutics [25, 68, 69].

Given the high costs of discovery, development, registration, and commercialization of novel therapeutic drugs, drug repositioning has attracted attention because of the well-known safety profiles of these drugs [54]. For example, terfenadine, commonly used for the treatment of auto-immune disorders such as allergic dermatitis, has been shown to prevent the secretion of VEGF from mast cells localized in the hypoxic microenvironment, and to induce ROS-mediated apoptosis and autophagy of malignant melanoma cells [70, 71]. Artemisinin and disease-modifying anti-rheumatic drugs can affect the response of cells to radiotherapy by regulating autophagy. Therefore, these drugs can be investigated as potential radiosensitizers [54].

### Glucose metabolism and radioresistance

Carbohydrates, the main source of cellular energy, mainly participate in the process of the oxidative decomposition of glucose, which comprises glycolysis and oxidative phosphorylation (OxPhos) [72]. In the 1920s, Warburg demonstrated that even in the presence of physiological oxygen levels, cancer cells can have active glycolytic phenotypes. This aerobic glycolysis is known as the Warburg effect, characterized by an increased glucose uptake rate, active glycolysis, and high lactic acid contents [73, 74]. Additionally, the synthesis of NADPH in cancer cells is induced through the pentose phosphate pathway (PPP) and the decrease in OxPhos levels in mitochondria, thereby reducing intracellular ROS levels and increasing tumor dependence on glycolysis [75]. Active glycolysis shows proliferative advantages during somatic cell carcinogenesis, and it represents an important component of malignant phenotype [76].

AKT is an important kinase that regulates various biological processes such as cell proliferation, survival, metabolism, and vascularization. AKT-mediated alterations in the cellular glucose metabolic pathway confer radioresistance to tumor cells when these cells are exposed to radiation for a long time [25]. The inhibition of mitochondrial respiration by mitochondrial respiratory modulators (e.g.*,* di-nitro phenol) leads to a considerable increase in the glycolytic index. The elevated glycolysis rate facilitates the rejoining of radiation-induced DNA strand breaks by activating both non-homologous end joining (NHEJ) and homologous recombination (HR) pathways, thus reducing the radiation-induced cytogenetic damage in cancer cells [77]. Additionally, radiotherapy can result in changes in many relevant molecules in the glycolytic pathway. In contrast, some key molecules in the glucose metabolism or its products, such as glucose transporter 1 (GLUT1), HIF1, and lactic acid, can affect the efficiency of radiotherapy [78–80].

#### GLUT1 role in radioresistance development

GLUT family represents a class of 14 proteins essential for glucose metabolism and found in the membranes of various cells. GLUT1 is the most common and widely distributed member of this family [78, 81], involved in the glucose transport and its expression is upregulated under hypoxic conditions, and therefore, it is often used as a cellular hypoxia marker [82]. GLUT1 overexpression was shown to be associated with the radioresistance and poor prognosis in oral squamous cell carcinoma and head and neck squamous cell carcinoma patients, which suggests that GLUT1 may be used as an indicator of the sensitivity to and prognosis of cancer radiotherapy [83, 84]. Radioresistant tumor cells often have high GLUT1 levels, which was associated with oncogene activation, tumor suppressor inactivation, stimulation of hypoxia, and the regulation of different signaling pathways, such as MAPK and PI3K/AKT [78].

**Fig. 2:**
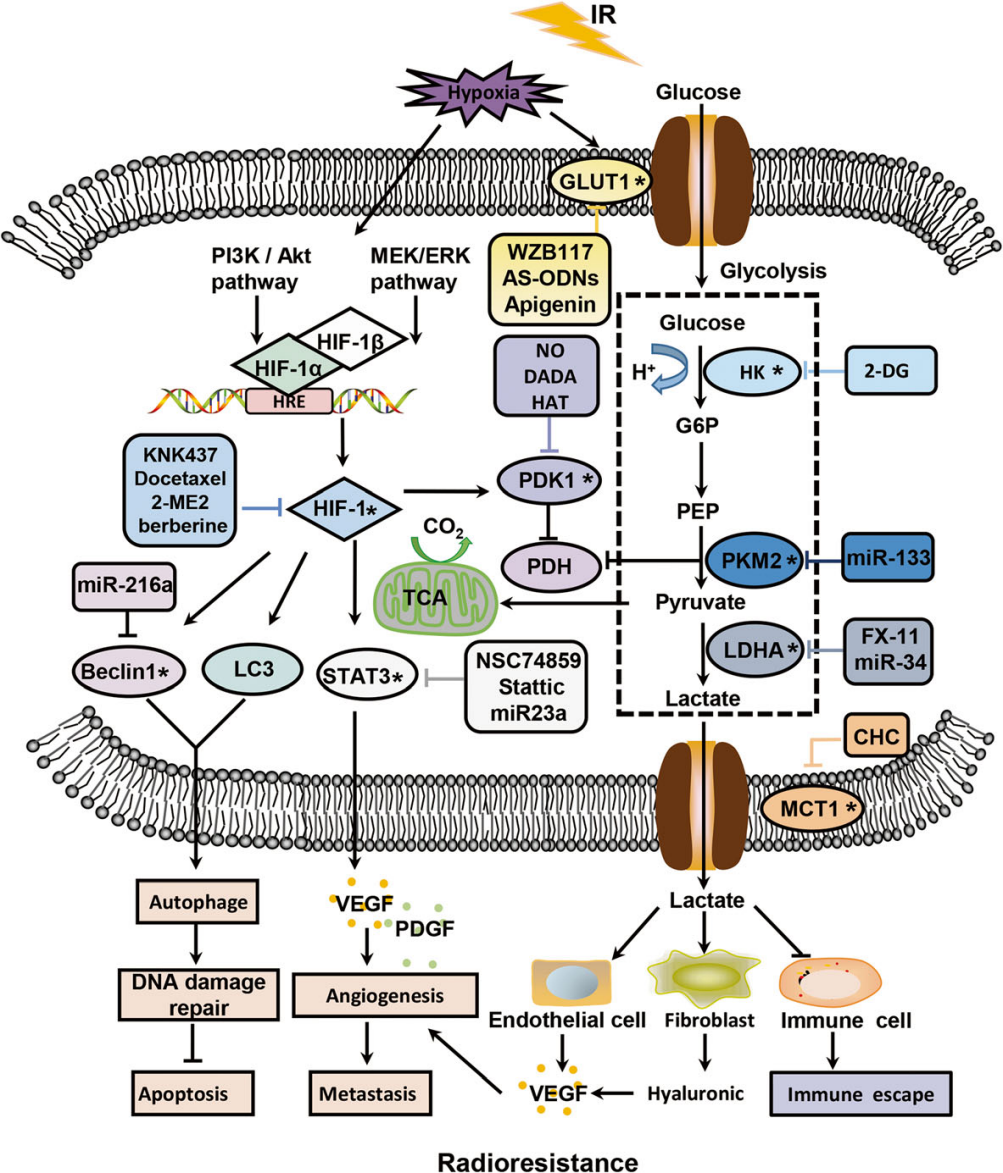
A schematic model illustrating the relationship between glucose metabolism and radiation resistance. Radiation-resistant cells exhibit an active glycolytic phenotype, and the enzymes in the glycolytic pathway play an important role in the process of radioresistance and can serve as targets for improving the efficacy of radiotherapy. In addition, HIF is able to activate glycolytic enzymes and promote the occurrence of radioresistance by inducing cell autophagy and angiogenesis. * was used to represent the targets to enhance radiosensitivity, the corresponding radiosensitizers are indicated in the same color in rectangle

Targeting GLUT1 and related signaling pathways may represent an effective way to improve radiotherapy efficacy [85]. As a natural flavonoid, apigenin was shown to have anti-proliferative and anti-angiogenic effects, exerted through the downregulation of GLUT1, HIF1α, and vascular endothelial growth factor (VEGF) expression [86]. Apigenin was confirmed to inhibit the expression of GLUT1 by regulating PI3K/AKT pathway, and improving the radiosensitivity of laryngeal carcinoma, prostate cancer, and adenoid cystic carcinoma cells [87–89]. Additionally, WZB117, a small molecule, acts as a specific inhibitor of GLUT1, overcoming the resistance of cancer cells to radiation [90]. WZB117 and radiation therapy combined can inhibit the growth of breast cancer cells and sensitize cancer cells to radiotherapy by increasing the level of intracellular ROS [91]. Furthermore, the antisense oligonucleotide chain (AS-ODNs) of GLUT1 can also induce the radiosensitivity of laryngeal carcinoma cells (Fig. 2) [92, 93]. Co-suppression of GLUT1 and the members of PI3K/AKT signaling pathway was shown to improve the radiosensitivity of laryngeal carcinoma xenograft cells in nude mice, suggesting that PI3K/AKT signaling pathway plays an important role in the development of radioresistance [94].

#### The role of lactic acid in radioresistance development

Lactic acid is one of the main products of glycolysis and one of the key factors in the development of malignant tumors. Alterations in glucose metabolism after radiotherapy can lead to the accumulation of large amounts of lactic acid, which is one of the unique malignant tumor phenotypes [26, 95]. The concentration of lactic acid in tumor tissues was shown to be significantly higher than that in the healthy tissues [96], and it can promote tumor metastasis, recurrence, and radioresistance, resulting in poor prognosis in many cancers [97]. Lactic content can predict metastasis rates, overall survival of patients, is a genotoxic stress biomarker, and it was shown to be associated with hypoxia-induced radioresistance [98]. In the nude mouse model of human squamous cell carcinoma, the accumulation of lactic acid was shown to correlate with radioresistance [80]. Michael et al. [99] demonstrated that the lactic acid accumulation induces many adverse effects in the cancer-associated stromal cells, which can help regulate angiogenesis by affecting proliferation, differentiation, and maturation of fibroblasts and endothelial cells. Lactic acid inhibits the activation and differentiation of many immune cells, such as dendritic and T-cells, by interfering with their metabolism and mediating immune escape [100, 101]. Additionally, it can induce the release of hyaluronic acid by tumor-associated fibroblasts, which promotes cell migration and clustering, VEGF secretion, and neovascularization [102]. All these represent potential mechanisms involved in the lactic acid-associated radioresistance (Fig. 2).

The monocarboxylate transporters (MCTs) can transport lactate through the cell membrane, and these molecules are encoded by the SLC16 gene family comprising 14 members, with only four subtypes (MCT1-MCT4) known to be proton transporters. Of these, MCT1 shows the highest affinity for the lactic acid [103]. Shiho et al. [97] showed that the levels of lactic acid in myeloma cells are associated with MCT1 expression. A competitive inhibitor of MCT1, CHC reduces the expression of lactic acid, inducing cell apoptosis (Fig. 2). The downregulation of MCT1 expression induces the expression of FAS protein in ovarian cancer cells, significantly inhibiting the activation of its downstream targets, such as FASL and FAP1, and promoting the expressions of apoptosis-related protein caspase-3, which indicates that MCT1 can induce resistance to cisplatin by antagonizing FAS (Fig. 2) [104], and that it may play the same role in radioresistance development.

Lactate dehydrogenase (LDHA) is found in almost all human tissues; it is a major enzyme catalyzing the conversion of pyruvate to lactic acid, and plays an important role in the glycolytic process [105]. Michael et al. [106–108] demonstrated that LDH5 overexpression can indicate hypoxic conditions, which can be associated with local recurrence, distant metastases, lower overall survival, and radioresistance of head and neck squamous cell carcinoma, prostate, and bladder cancers. Furthermore, soluble adenylate cyclase (sAC) promotes the release of LDHA, accelerates cell proliferation, and induces the anti-irradiation effects in prostate cancer cells through the activation of BRAF/ERK1/2 signaling pathway [109]. FX-11, a specific inhibitor of LDHA, can promote the generation of DSBs and cell apoptosis by reducing the EMT, DNA repair capacity, hypoxia, and autophagy in prostate cancer cells, improving cell sensitivity to radiotherapy [105]. Acting as a tumor suppressor, the expression of miR-34 was shown to negatively correlate with radioresistance development and to induce the sensitivity of the hepatocellular carcinoma cells to radiotherapy by inhibiting the expression of LDHA (Fig. 2) [110, 111].

#### HIF1 and radioresistance

Hypoxic conditions in cells often have a negative impact on the radiotherapy outcomes [112]. Tumors rely more on anaerobic glycolysis for energy production than the normal tissues. Malignant tumor environments are often hypoxic, and they rely on exacerbated glycolysis to meet the increased demand for ATP and biosynthetic precursors [113]. Hypoxic environments promote the transformation of tumor cell metabolism from the oxidative metabolism to anaerobic glycolysis, which protects tumor cells and induces the development of radioresistance in tumor stem cells [114]. In human osteosarcoma cells, hypoxia was shown to confer anti-irradiation effects and induce the expression of autophagy-related proteins LC3 and LC3-II, suggesting that hypoxia can activate cell autophagy and accelerate the removal of ROS from the cells, leading to radioresistance development [115]. Moreover, hypoxia can activate EGFR and NRF2 expression in lung cancer cells, inducing radioresistance (Fig. 2) [116], together with the activation of *HIF1* transcription, which regulates the adaptive cellular responses to hypoxia [112]. HIF1 has been identified as an important mediator of the carbohydrate metabolic pathway reprogramming from OxPhos to glycolysis [117], and it is composed of two subunits, HIF1α and HIF1β, and HIF1α expression is oxygen-dependent due to the presence of an oxygen-dependent degradation domain. HIF1β, known as aryl hydrocarbon receptor nuclear translocator (ARNT), is constitutively expressed, and therefore, not affected by environmental conditions [118]. The heterodimer formed by HIF1α/HIF1β constitutes a functional HIF1 molecule, characterized by the presence of hypoxia-responsive element within the promoter or enhancer region [119]. HIF1α is the main regulatory subunit of this molecule, which binds to the promoter and upregulates HIF1β expression [120]. A previous study confirmed that HIF1α can activate the transcription of target genes that regulate various biological processes, including cell proliferation, glucose metabolism, and pH regulation, playing a vital role in the adaptation of cancer cells to hypoxic conditions (Fig. 2) [121].

A number of studies demonstrated that HIF1 promotes tumor invasion, metastasis, and mediates the anti-irradiation effects [112]. The mechanisms underlying the development of radioresistance may include the following:

1. The promotion of tumor angiogenesis. HIF1 activates the expression of angiogenic cytokines such as VEGF and platelet-derived growth factor (PDGF), enabling radioresistance development in endothelial cell and increasing tumor vascular proliferation and regrowth (Fig. 2) [122].
2. Inhibition of apoptosis. N-Myc downstream regulatory gene 2 (NDRG2) is a downstream target of HIF1, which inhibits the expression of pro-apoptotic protein BAX, promoting the development of radioresistance. HIF1α can also directly inhibit p53-induced apoptosis, and the p53 status is a major determinant of the HIF1 effects on tumor radiosensitivity [123, 124].
3. Activation of radioresistance-related signaling pathways. HIF1α can induce the expression of CXCL8, a chemokine with tumorigenic and angiogenic roles. CXCL8 expression further activates AKT/mTOR/STAT3 signaling pathway, supporting liver cancer progress and metastases and inducing radiotherapy resistance [125]. Additionally, the MEK/ERK signaling transduction pathway mediates the sustained expression of DNA protein kinase (DNA-PKcs), which regulates the expression and activity of HIF1 protein, thereby inducing radioresistance in glioblastomas [126].
4. Hypoxia-induced autophagy. Autophagy is activated in response to stress (e.g.*,* hypoxic conditions), and HIF1α was shown to be involved in the radiation-induced autophagic cell death in breast cancer cells. Elevated autophagy levels reduce the IR-induced DNA damage [127]. Sun et al. [128] found that hypoxia-induced autophagy can lead to the resistance of colon cancer cells to radiotherapy through the activation HIF1/miR-210/BCL2 pathway. High HIF1 levels can trigger autophagy activation and induce the expression of autophagy-associated protein LC3 and the degradation of p62. Furthermore, HIF1α can increase the phosphorylation of c-JUN, a downstream HIF1α molecule, and the expression of autophagy gene BECN1, which mediates radioresistance in lung cancer cells (Fig. 2) [129].
5. Hypoxia-induced CSC activation. A hypoxic or perinecrotic microenvironment was found to be advantageous for the survival and proliferation of other types of CSCs. Hypoxic cells express higher levels of CSC markers such as CD44, CD133, OCT3/4, and SOX2 [130, 131]. CD44 promotes CSC phenotype and resistance to radiation [132], and its isoforms, the standard isoform, CD44s, and several variant isoforms (CD44v), have different functions. Following the irradiation, CD44s expression is strongly upregulated in a dose-dependent manner, compared with that of CD44v, and contributes to the longer-term cell survival by maintaining ERK phosphorylation and the radiation-induced EMT [133]. CD44v is produced by alternative splicing regulated primarily by ESRP1/2 and has recently been shown to stabilize anti-ROS machinery by stabilizing xCT (cysteine/glutamate antiporter) on the cell membrane [134]. Anticancer therapy leads to the ectopic expression of CD44v in osteosarcoma and hepatic cancer cells of patients with Li-Fraumeni disease [135]. This may be because an undetectable number of CD44v8–10-positive CSCs produces excess ROS levels due to radiotherapy and chemotherapy. A mutually exclusive expression pattern of CD44v8–10 and c-MYC was observed based on the activation of the ROS-mediated β-catenin/Wnt signaling pathway [136, 137]. The ubiquitin ligase Fbw7 family regulates c-Myc expression, exerting the anti-tumor effect [138]. CD44 has been reported as a useful marker for the prediction of tumor radiosensitivity, and its levels can be used for the prediction of local recurrence after laryngeal cancer radiotherapy [139]. Decreased levels of ROS and apoptosis in CD44^+^ CD24^+^ cells may contribute to the development of radioresistance in pancreatic cancer [140]. Radical cystectomy is preferred for the cases where the overexpression of CD44 and/or IL6 is observed in the preoperative specimens [132]. Both HIF1 and HIF2α are activated under hypoxic conditions and promote the stem-like properties of cancer cells. Furthermore, HIF2α was recently shown to contribute together with the intracellular domain of CD44 generated by γ-secretase to the acquisition of radioresistance by glioma stem cells in a perivascular niche rich in osteopontin [141].

An increasing number of studies have shown that, by targeting HIF1 activity, tumor antioxidant capacity can be reduced, as it affects the TME and promotes the sensitivity of solid tumors to radiotherapy. By combining HIF1 targeting and radiotherapy, improved therapeutic effects can be achieved [142]. For example, the use of chetomin, a chemical HIF1α inhibitor, can disrupt the interactions between this molecule and p300, attenuate hypoxia-induced gene expression, and increase radiosensitivity of cancer cells under severe hypoxic conditions [143]. Additionally, HIF1α inhibition leads to the downregulation of stem cell markers and a decrease in radioresistance of cervical cancer cells [144]. Various HIF1 inhibitors function through different signaling pathways, thereby enhancing the efficacy of radiotherapy. KNK437 is a benzylidene lactam compound that inhibits the synthesis of heat shock proteins (HSPs), which increase DNA damage repair and inhibit cell death, stabilizing HIF1α expression and promoting radioresistance. KNK437 can abrogate hypoxia-induced anti-radiation effects by targeting both AKT and HIF1α [145]. Furthermore, 2-methoxyestradiol is an estrogen metabolite that suppresses HIF1α levels and its transcriptional activity. It depolymerizes microtubules and prevents HIF1α nuclear accumulation [146], increasing the radiosensitivity of NPC stem cells and melanoma cells by inactivating NF-κB/HIF1 or HIF1α/PDK1 signaling pathway [147, 148]. Berberine can inhibit tumor metastasis, tumorigenicity, and growth, and transforming growth factor-β (TGF-β)-induced tumor invasion and EMT [149]. Moreover, it enhances the radiosensitivity of NPC cells by inhibiting the expression of HIF1α and VEGF [150]. NVP-BEZ235, an inhibitor of PI3K/mTOR signaling pathway, can inhibit the activation of HIF1α/VEGF signaling pathway in endometrial cancer and suppress radioresistance development [151]. As STAT3 inhibitors, NSC74859 and Stattic can improve the radiosensitivity of esophageal cancer through the inhibition of hypoxia and radiation-induced STAT3 activation, as well as the expression of HIF1α and VEGF [152, 153]. Additionally, docetaxel is a semi synthetic paclitaxel derived from European yew, which has been widely applied in the clinical treatment of gastric, non-small cell lung (NSCL), ovarian, and breast cancers [154], and which was shown to induce the activation of JNK2 signaling pathway, mediate the phosphorylation of PHD1, and inhibit the expression of HIF1α, leading to the apoptosis of the tumor cells in hypoxic conditions [155]. Finally, paclitaxel pretreatment was shown to inhibit the radioresistance of HIF1α-induced hepatocellular carcinoma and lung adenocarcinoma, suggesting that it can be used as a sensitizer for radiotherapy (Fig. 2) [156].

In addition to the inhibitors of HIF1, many miRNAs can promote or inhibit hypoxia-induced radioresistance. MiR-210 was shown to be expressed in the different types of tumor and normal cells in hypoxic environments [157]. The expression of miR-210 promotes the DSB repair, increases the production of lactic acid, and HIF1α stability [158]. The downregulation of miR-210 significantly inhibits cell viability, inducing G0/G1 phase cell cycle arrest, and increasing apoptosis rates and the radiosensitivity of hypoxic hepatocarcinoma cells [157, 159]. Furthermore, miR-21 was shown to regulate the radiosensitivity of cervical cancer cells through the PTEN/AKT/HIF1α feedback loop and the AKT-mTOR signaling pathway [160]. Hypoxia-responsive miR-124 and miR-144 overexpression can inhibit hypoxia-induced autophagy and enhance the radiosensitivity of prostate cancer cells by downregulating the expression of the PIM1 oncogene [161], while miR-216a can enhance the radiosensitivity of pancreatic cancer cells by inhibiting BECN1-mediated autophagy (Fig. 2) [162]. Additionally, a decrease in miR-23a expression promotes the radioresistance of NPCs, determining their response to radiotherapy [163].

#### The role of other molecules associated with glucose metabolism in radioresistance development

Pyruvate kinase (PK) can convert phosphoenolpyruvate and ADP into pyruvate and ATP, which makes it one of the major rate-limiting enzymes in glycolysis. A previous study demonstrated that the PK expression positively correlates with the radiotherapy resistance in tumor cells [164]. The M2 isoform (PKM2) is a key regulator of glycolysis, expressed only in cancer cells [165], and targeting this molecule can inhibit cell viability, induce G2/M arrest, and promote apoptosis. Additionally, this can increase the radiosensitivity of NSCLCs and the IR-induced apoptosis and autophagy rates, which are associated with the inhibition of AKT and PDK1 phosphorylation [166]. PKM2 is targeted by miR-133 as well, and this miRNA is downregulated in radioresistant lung cancer cells. MiR-133b resensitizes radioresistant lung cancer cells by inhibiting PKM2-mediated glycolysis [167]. Nitric oxide (NO) levels were shown to be significantly associated with cellular metabolism, and a decrease in the NO levels leads to a significant reduction in PDK1 expression, enhancing the radiosensitivity of hypoxic NSCLCs [168]. Furthermore, dichloroacetate, a PDK inhibitor, can effectively radiosensitize glioblastoma cells [164], while the treatment of esophageal squamous cell carcinoma cells with diisopropylamine dichloroacetate (DADA) can increase their sensitivity to radiation (Fig. 2) [169].

Hexokinase 2 (HK2) is a key glycolytic enzyme in glucose metabolism, highly expressed in a variety of human solid tumors. Its upregulation can induce glycolysis, and it is essential for tumor progression and maintenance. By inhibiting HK2 signaling in cancer cells, their radiosensitivity may increase [170]. Additionally, 2-deoxy-D-glucose (2-DG) is an inhibitor of glucose metabolism and ATP production, which can help suppress the IR-induced radioresistance [171]. Following the phosphorylation of HK2 by 2-DG, this molecule can disrupt the radiation-induced DNA damage repair in tumor cells and promote their apoptosis by reducing intracellular energy levels [172]. The combination of 2-DG and histone deacetylase transferase inhibitors can induce apoptosis in glioblastoma cells [173], while 2-DG can also significantly inhibit the expression of HK2 and induce apoptosis (Fig. 2) [174].

##### Mitochondrial metabolism and radiotherapy resistance

In addition to glucose metabolism, mitochondrial metabolism is closely related to radioresistance development as well. Mitochondria exist in most cells and are the main sites of cellular aerobic respiration, with different energy metabolic pathways. Mitochondria adapt to the rapid tumor growth requirements by regulating the energy production process [175]. Radiotherapy resistance in cancer cells is associated with changes in the mitochondrial energy metabolism, mitochondrial size, morphology, and functions. Furthermore, mitochondrial mutation rate, respiration, and intracellular ATP levels are increased as well [176]. Mitochondrial membrane potential (MMP) and the expression of several proteins involved in mitochondrial energy metabolism play important roles in tumor radiotherapy resistance.

#### Mitochondrial oxidative stress and radioresistance

**Fig. 3:**
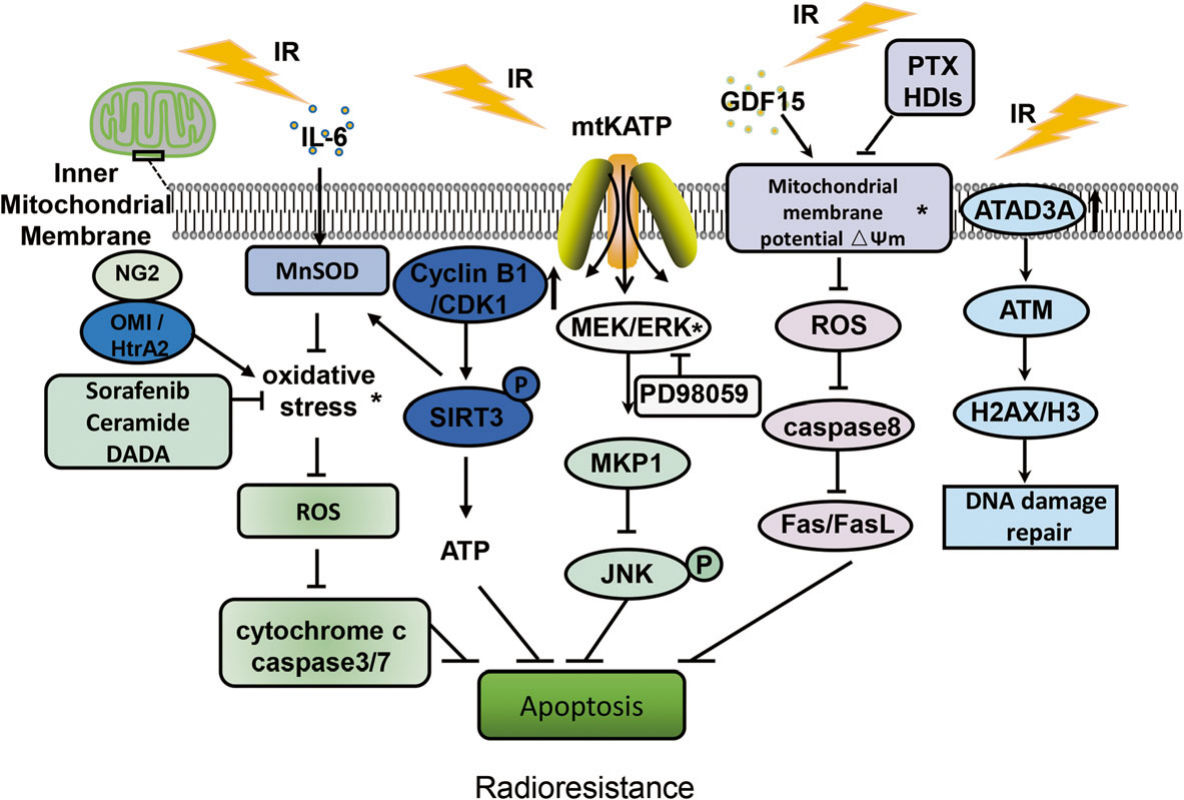
A schematic model illustrating the relationship between mitochondrial metabolismand radiation resistance. IR inhibits the oxidative stress of mitochondria, causes abnormal expression of mitochondrial protein and increases the mitochondrial membrane potential, thereby promoting DNA damage repair and inhibiting apoptosis, leading to the occurrence of radioresistance. * was used to represent the targets to enhance radiosensitivity, the corresponding radiosensitizers are indicated in the same color in rectangle

The redox environment, representing a balance between the generation of ROS species and their removal by antioxidant enzymes, is a key regulator of oxidative stress in cells. Excessive superoxide levels, if not removed by endogenous antioxidants or related enzymes, can cause oxidative stress damage in mitochondria. Manganese superoxide dismutase (MnSOD) is the main antioxidant enzyme, which can catalyze the disproportionation of superoxide anion radicals, protecting the body from ROS-induced damage. Moreover, MnSOD regulates the response of cells, tissues, and organs to the IR, which is essential for the protection of mitochondria and cells from oxidative stress (Fig. 3). The increased activity of MnSOD was shown to increase significantly the viability of pancreatic cancer cells after γ-ray irradiation and activate G2 checkpoint block, ultimately inducing radioresistance in pancreatic cancer cells [177].

Many studies demonstrated that the protection of cells against the mitochondrial oxidative stress-induced cell death can promote radioresistance development. Maus et al. [178] showed that the glial cell antigen 2 (NG2) can protect oligodendrocyte precursor cells from oxidative stress by interacting with mitochondrial serine protease OMI/HtrA2. NG2 downregulation can increase the apoptosis and cell sensitivity to oxidative stress. This interaction between NG2 and OMI/HtrA2 may contribute to radioresistance development in gliomas (Fig. 3). Additionally, following treatment with Il6, rat glioma cells developed radioresistance by suppressing mitochondrial oxidative stress. Therefore, Il6 treatment induces radioresistance in tumor cells by inhibiting the increase in ROS levels (Fig. 3) [179].

In contrast, enhanced mitochondrial oxidative stress can promote tumor cell radiosensitivity. Sorafenib is a novel anti-cancer drug that can induce the apoptosis of drug- and radiation-resistant hepatoma cells through mitochondrion-dependent oxidative stress mechanisms. The mechanism of its action includes rapid formation of ROS in mitochondria, triggering of the mitochondrial calcium overload, and activating apoptotic processes by releasing cytochrome C and activating caspase 3/7 pathway [180]. Ceramides induce ROS accumulation as well, through the activation of mitochondrial/caspase apoptosis pathways, and inhibiting radioresistance [181]. Moreover, DADA regulates the switch from glycolysis to OxPhos and induces intracellular ROS level increase, thereby enhancing the radiosensitization of esophageal squamous cell carcinoma (Fig. 3) [169].

#### Mitochondrion-associated protein and radioresistance

The mechanisms underlying cell irradiation are complex, and they are involved in the inhibition of cell proliferation and induction of cancer cell apoptosis. Mitochondrial proteins are involved in apoptosis as well, and therefore, they may play a key role in the radiation signal transduction. The mitochondrial proteomes derived from Burkitt lymphoma before and after irradiation were analyzed and 23 differentially expressed proteins were identified [182]. This suggests that radiotherapy can lead to the considerable alterations in the mitochondrial protein expression, and therefore induce radioresistance.

Currently, the mitochondrion-associated proteins that were shown to be associated with radioresistance include the following: (1) Adenosine monophosphate family protein 3A (ATAD3A), often expressed in cancer patients and shown to be related to the sensitivity of cancer patients to chemotherapeutics. Increased ATAD3A expression can inhibit the IR-induced apoptosis in glioblastoma cells. After silencing ATAD3A, the expression of ATM, histone H2AX, and H3 was shown to decrease, inhibiting the DNA damage repair and ultimately promoting tumor cell radiosensitivity [183]. (2) NAD^+^-dependent protein deacetylase SIRT3 is a member of the sirtuin family, present in mitochondria, that promotes metabolic homeostasis by modulating mitochondrial protein deacetylation. Following the *SIRT3* transcription, cyclin B1/CDK1 further induce SIRT3 activity. Mutations in the SIRT3 Thr150Ala/Ser159Ala lead to a decrease in MnSOD activity and the production of mitochondrial ATP, increasing sensitivity to radiotherapy. Therefore, the targeting of CDK1-mediated phosphorylation of SIRT3 may represent an effective way to sensitize tumor cells to radiation therapy [28]. (3) Mitochondrial MAPK phosphatase (MKP1) represents a potential target for the treatment of human epidermal growth factor receptor 2 (HER2)-positive breast cancers. MKP1 is overexpressed in radioresistant breast cancer cells and it can translocate to mitochondria after irradiation, preventing the activation of apoptosis by inhibiting the accumulation of phosphorylated JNK. MKP1 is the major downstream effector of the HER2-activated RAF/MEK/ERK1/2 pathway. Mitochondrial MKP1 confers radioresistance to HER2 overexpressing breast cancer cells, and by co-suppressing the expression of MKP1 and HER2, breast cancer cell apoptosis can be induced, while inhibiting radioresistance (Fig. 3) [184].

#### MMP and radioresistance

During respiratory oxidative processes, the MMP is generated due to the asymmetric distribution of protons and other ions on both sides of the inner mitochondrial membrane. A physiological MMP is a prerequisite for the maintenance of the physiological cell functions. Studies have shown that some molecules and related signaling pathways induce radioresistance by increasing MMP or inhibiting its decrease. Therefore, targeting the MMP can be an effective way to increase radiosensitivity. The main molecules and processes underlying MMP effects on radiosensitivity include the following: (1) Growth differentiation factor-15 (GDF15), a member of the TGF-β superfamily, participates in homeostasis maintenance and regulates radiosensitivity. Li et al. [185] demonstrated that GDF15 contributes to radioresistance development in head and neck cancer (HNC) cells by activating MMP and inhibiting intracellular ROS generation. Therefore, GDF15 levels may indicate radioresistance levels and its expression may represent a potential therapeutic target for the treatment of HNC. (2) MEK/ERK-mediated signaling selectively inhibits IR-induced decrease in MMP and inhibits FAS-mediated cell death by inhibiting caspase-8 activity. MEK-specific inhibitor PD98059 was shown to prevent the observed effects of MEK/ERK on MMP and the development of radioresistance [186]. (3) Histone deacetylase inhibitors increase radiosensitivity by reducing MMP and promoting ROS production, G2/M phase arrest, and the IR-induced apoptosis of the esophageal cancer cells [187]. (4) In the radiation-resistant cells (CRR) treated with paclitaxel, ROS levels can increase through the decrease in MMP and OxPhos activation, thus overcoming the radioresistance of these cells (Fig. 3) [188].

Furthermore, mitochondrial ion channels are involved in radioresistance development as well. Different types of ion channels can be found on the inner and outer mitochondrial membranes, and they are involved in many important cellular processes, including ATP production, apoptosis, and cell proliferation [189]. Some ion channels as therapeutic targets have been widely used in clinic. Mitochondrial KATP channel (mtKATP channel) is an important member of this family. MtKATP overexpression was found to be closely related to the degree of malignancy of gliomas and the overall survival of patients. Importantly, mtKATP channel can regulate glioma radioresistance development by modulating the ROS-induced ERK activation (Fig. 3), suggesting that the mtKATP pathway is a key regulator of radiosensitivity in gliomas, and the blockers and inhibitors of mtKATP channel and MAPK/ERK kinase, respectively, may represent novel therapeutics of the treatment of gliomas [190].

## Conclusions

Radioresistance emerged as one of the major obstacles to cancer treatment, and it is caused by numerous factors. An increasing number of studies demonstrated that radioresistance development can be associated with tumor metabolism, as the radiotherapy may induce alterations in many molecules and signaling pathways involved in the tumor cell metabolism, and metabolic changes may affect the efficacy of radiotherapy. Therefore, previous studies investigated the changes in glucose, mitochondrial, and other metabolic processes, and the effects of these changes on cellular radioresistance. Based on these results, many molecules or inhibitors were developed, as shown in Table 1, which can target specific metabolic processes or molecules, to be used as radiotherapy sensitizers for the inhibition of radioresistance development in tumors. However, these sensitizers induce side effects in many non-cancerous cells, since they do not show high specificity and efficacy. Taken together, the mechanisms underlying the development of radioresistance should be further studied, together with the roles of tumor metabolism in these processes, in order to identify novel, more efficient and specific radiosensitizers, and provide novel strategies for the treatment of malignant tumors.

**Table 1:** Metabolism-associated targets in radioresistance and the radiosensitization methods

Items	Targets	Radiosensitizer	Reference
Glycose metabolism	GLUT1	Apigenin,WZB117	[77, 81]
	MCT1	CHC	[94]
	LDHA	FX-11, miR-34	[95, 100, 101]
	PKM2	miR-133, DADA	[145, 147]
	HK2	2-DG	[149, 150]
	HIF	Chetomin, KNK437, 2-ME2,	[121, 123, 124]
		Barberin, NVP-BEZ235, miR-216a	[128, 129, 140]
		NSC74859, Stattic, Docetaxel	[130–133]
		miR-21, miR-124, miR-144	[138, 139]
Mitochondrial metabolism	Oxidative stress	Sorafenib, Ceremides, DADA	[147, 158, 159]
	MMP	PD98059, HDIs, Paclitaxel	[164–166]

## Abbreviations

2-DG: 2-deoxy-D-glucose
ARNT: Aryl hydrocarbon receptor nuclear translocator
AS-ODN: Antisense oligonucleotide chain
ATAD3A: Adenosine monophosphate family protein 3A
CSC: Cancer stem cell
DDR: DNA damage response
DSB: Double-strand break
EMT: Epithelial-mesenchymal transition
GBM: Glioblastoma multiforme
GDF15: Growth differentiation factor-15
GLUT1: Glucose transporter 1
HIF1: Hypoxia inducible factor 1
HK2: Hexokinase 2
HNC: Head and neck cancer
HR: Homologous recombination
HSP: Heat shock protein
IGF: Insulin-like growth factor
IR: Ionizing radiation
LDHA: Lactate dehydrogenase
MAPK: Mitogen-activated protein kinase
MCT: Monocarboxylate transporter
MMP: Mitochondrial membrane potential
MnSOD: Manganese superoxide dismutase
MRD: Minimal residual disease
NDRG2: N-Myc downstream regulatory gene 2
NG2: Glial cell antigen 2
NHEJ: Non-homologous end joining
NO: Nitric oxide
NPC: Nasopharyngeal carcinoma
NRF2: Erythroid 2-related factor 2
NSCL: Non-small cell lung
OxPhos: Oxidative phosphorylation
PDGF: Platelet-derived growth factor
PI3K: Phosphatidylinositol 3-kinase
PK: Pyruvate kinase
PPP: Pentose phosphate pathway
ROS: Reactive oxygen species
sAC: Soluble adenylate cyclase
SSB: Single-strand break
TGF-β: Transforming growth factor-β
TME: Tumor microenvironment
TMZ: Temozolomide
VEGF: Vascular endothelial growth factor
